# Growth Dynamics and Survival of *Liberibacter crescens* BT-1, an Important Model Organism for the Citrus Huanglongbing Pathogen “*Candidatus* Liberibacter asiaticus”

**DOI:** 10.1128/AEM.01656-19

**Published:** 2019-10-16

**Authors:** Marta Sena-Vélez, Sean D. Holland, Manu Aggarwal, Nick G. Cogan, Mukesh Jain, Dean W. Gabriel, Kathryn M. Jones

**Affiliations:** a Department of Biological Science, Florida State University, Tallahassee, Florida, USA; b Department of Mathematics, Florida State University, Tallahassee, Florida, USA; c Department of Plant Pathology, University of Florida, Gainesville, Florida, USA; University of Illinois at Chicago

**Keywords:** *Liberibacter crescens*, “*Candidatus* Liberibacter asiaticus,” Huanglongbing, citrus greening, growth dynamics, viable but not culturable, alkalinization, ammonia production, organic acid consumption

## Abstract

Liberibacter crescens is the only cultured member of its genus, which includes the devastating plant pathogen “*Candidatus* Liberibacter asiaticus,” associated with citrus greening/Huanglongbing (HLB). *L. crescens* has a larger genome and greater metabolic flexibility than “*Ca*. Liberibacter asiaticus” and the other uncultured plant-pathogenic *Liberibacter* species, and it is currently the best model organism available for these pathogens. *L. crescens* grows slowly and dies rapidly under current culture protocols and this extreme fastidiousness makes it challenging to study. We have determined that a major cause of rapid death of *L. crescens* in batch culture is its alkalinization of the medium (to pH 8.5 by the end of logarithmic phase). The majority of this alkalinization is due to consumption of alpha-ketoglutaric acid as its primary carbon source, with a smaller proportion of the pH rise due to NH_3_ production. Controlling the pH rise with higher buffering capacity and lower starting pH improved recoverability of cells from 10-day cultures by >1,000-fold. We have also performed a detailed analysis of *L. crescens* growth with total cell numbers calibrated to the optical density and the percentage of live and recoverable bacteria determined over 10-day time courses. We modified *L. crescens* culture conditions to greatly enhance survival and increase maximum culture density. The similarities between *L. crescens* and the pathogenic liberibacters make this work relevant to efforts to culture the latter organisms. Our results also suggest that growth-dependent pH alteration that overcomes medium buffering should always be considered when growing fastidious bacteria.

**IMPORTANCE** Liberibacter crescens is a bacterium that is closely related to plant pathogens that have caused billions of dollars in crop losses in recent years. Particularly devastating are citrus losses due to citrus greening disease, also known as Huanglongbing, which is caused by “*Candidatus* Liberibacter asiaticus” and carried by the Asian citrus psyllid. *L. crescens* is the only close relative of “*Ca*. Liberibacter asiaticus” that can currently be grown in culture, and it therefore serves as an important model organism for the growth, genetic manipulation, and biological control of the pathogenic species. Here, we show that one of the greatest limitations to *L. crescens* growth is the sharp increase in alkaline conditions it produces as a consequence of consumption of its preferred nutrient source. In addition to new information about *L. crescens* growth and metabolism, we provide new guidelines for culture conditions that improve the survival and yield of *L. crescens*.

## Abstract (precis)

Liberibacter crescens is a bacterium that is closely related to plant pathogens that have caused billions of dollars in crop losses in recent years. Particularly devastating are citrus losses due to citrus greening disease, also known as Huanglongbing, which is caused by “*Candidatus* Liberibacter asiaticus” and carried by the Asian citrus psyllid. *L. crescens* is the only close relative of “*Ca*. Liberibacter asiaticus” that can currently be grown in culture, and it therefore serves as an important model organism for the growth, genetic manipulation, and biological control of the pathogenic species. Here, we show that one of the greatest limitations to *L. crescens* growth is the sharp increase in alkaline conditions it produces as a consequence of consumption of its preferred nutrient source. In addition to new information about *L. crescens* growth and metabolism, we provide new guidelines for culture conditions that improve the survival and yield of *L. crescens*.

## INTRODUCTION

The citrus greening/Huanglongbing (HLB) bacterium “*Candidatus* Liberibacter asiaticus” is the most devastating citrus pathogen in history (1–3). Like many other bacteria that live in the phloem vessels of plants, “*Ca*. Liberibacter asiaticus” cannot yet be grown in pure culture (axenic medium) (4–6). Liberibacter crescens is the sole member of this genus that can be grown in axenic media (7, 8), and it has become a model organism for study of the plant-pathogenic liberibacters. These pathogens include “*Ca*. Liberibacter americanus” and “*Ca*. Liberibacter africanus,” which also cause citrus greening, and “*Ca*. Liberibacter solanacearum,” which attacks tomato and other plants of the family Solanaceae and plants of the family Apiaceae or Umbelliferae (9, 10). The inability to culture the *Liberibacter* pathogens makes the study of these bacteria very challenging (10). *L. crescens* is a nonpathogenic species that has been isolated from the environment only once, from Babaco papaya in Puerto Rico (7, 8). Although “*Ca*. Liberibacter asiaticus” has a significantly reduced genome relative to *L. crescens* (1.23 Mb versus 1.5 Mb), the predicted functions encoded in the genomes have significant overlap, including genes involved in central metabolism (8). Study of the culture dynamics and metabolism of *L. crescens* may provide clues to critical factors required for growth and culture of other *Liberibacter* species, including “*Ca*. Liberibacter asiaticus.” *L. crescens* is quite fastidious; it is slow growing, and only three medium formulations for its culture have been described (7, 8, 11). We observed that within a very short time after reaching stationary phase in batch culture, *L. crescens* cells become unrecoverable when transferred to fresh medium. This death phase appears to be much more rapid than that of related alphaproteobacteria, such as Sinorhizobium meliloti (12, 13). In this study, we determined a major cause of *L. crescens* BT-1 culture death and further investigated the factors leading to death. We also performed a detailed characterization of the growth dynamics of time course cultures of this little-understood organism. Our results and improvements to culture protocols will greatly facilitate the study of *L. crescens* and may provide insights into the obstacles to culture of the *Liberibacter* plant pathogens.

## RESULTS

### *L. crescens* BT-1 rapidly loses viability in stationary phase in BM7 medium.

**FIG 1:**
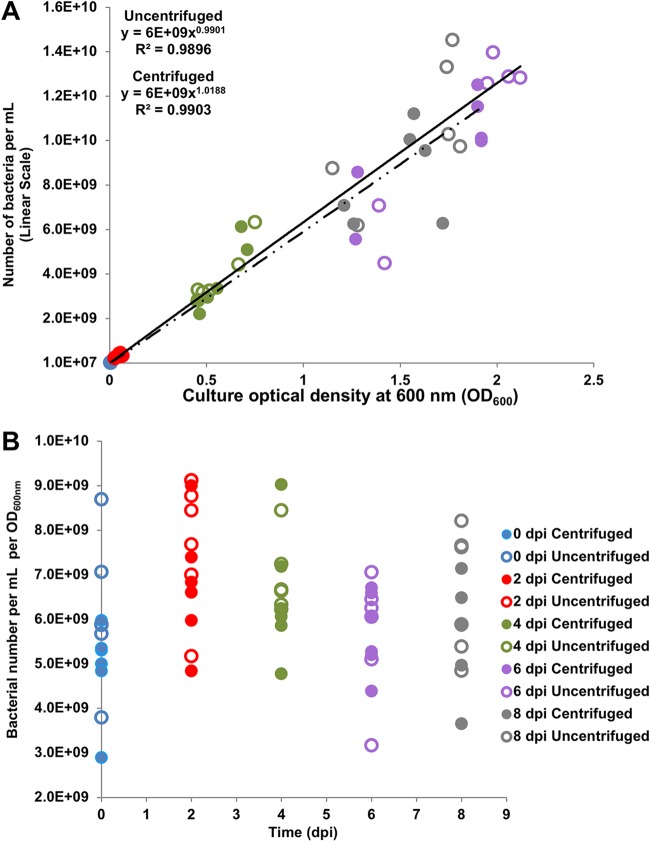
Number of bacteria *per* OD_600_ in Liberibacter crescens cultures. (A) Relationship between *L. crescens* culture optical density (OD_600_, *x* axis) and the number of bacteria counted on a hemocytometer grid (*y* axis). Each time point is shown in a different color (see legend). Aliquots that were centrifuged and suspended in 0.15 M NaCl before diluting and counting are represented by solid circles. Aliquots that were counted without centrifugation are represented by empty circles. The trend line from three different experiments with two technical replicates each shows the best fit to a power function (equations shown on graph). This allowed us to determine that 1 OD_600_ unit is equivalent to an average of 6E^09^ bacteria ml^−1^. (B) Bacterial number per OD_600_ unit for every replicate performed and at all time points.

The extremely rapid death of *L. crescens* in stationary phase led us to perform a detailed analysis of the growth of this bacterium in batch culture. In this study, the total cells (P_total_), live cells (P_live_), and recoverable cells (P_R_) were quantified at each stage of growth and in different growth conditions. (See Materials and Methods for detailed definitions of each population.) To establish baseline values, both the optical density at 600 nm (OD_600_) of the culture and the number of bacterial cells per ml counted on a hemocytometer grid were determined every other day over 8 days of growth at 29 to 30°C (Fig. 1). *L. crescens* was grown in BM7 (7), a complex rich medium containing fetal bovine serum (FBS), Grace’s insect medium (TNM-FH; HiMedia), alpha-ketoglutaric acid (αkg), ACES [*N*-(2-acetamido)-2-aminoethanesulfonic acid] buffer, and potassium hydroxide. (See Materials and Methods.) Both centrifuged/resuspended and uncentrifuged culture aliquots were counted to determine whether cell ghosts that resist sedimentation make a significant contribution to the hemocytometer counts, especially in late stationary phase when cell lysis may occur. There were few differences in the number of total counted cells in the centrifuged versus the uncentrifuged bacterial aliquots (Fig. 1; see also Fig. S1A and B in the supplemental material). Using these data, we established that one optical density unit at OD_600_ is equivalent to 6E^09^ *L. crescens* BT-1 cells per ml (Fig. 1). The numbers of cells ml^−1^ per OD_600_ unit were similar at all growth stages (Fig. 1B and Fig. S1C and D). These data were used to calculate the total cell population (P_total_) in subsequent experiments. By quantifying the total cell number ml^−1^ per OD_600_ unit, we established a framework for determining the fraction of the total population that is viable and the fraction of the total that can reestablish growth after plating on BM7.

**FIG 2:**
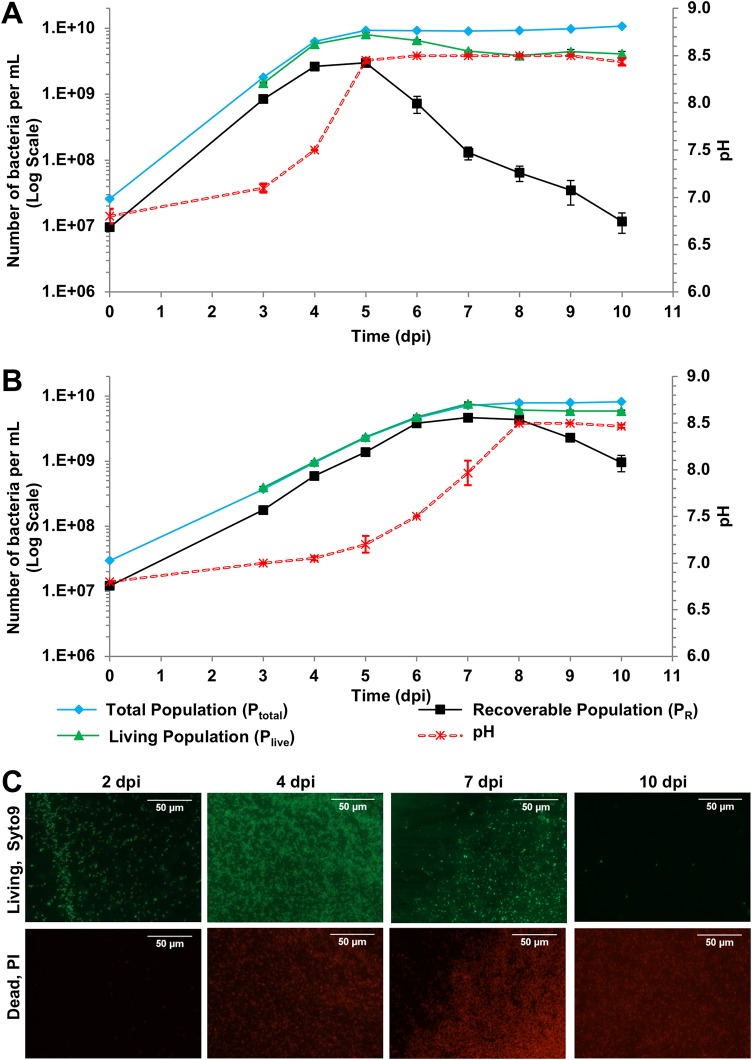
*L. crescens* growth curves and pH increase during growth. (A and B) The different bacterial populations present in *L. crescens* cultures and the number of bacteria (left *y* axis) in each population in cultures incubated at 29 to 30°C (A) and room temperature (B). The total population, P_total_, is shown in blue, the P_live_ is shown in green, and the recoverable P_R_ is shown in black. The pH (secondary *y* axis, right) increase of the cultures during the time course is shown as a dashed red line. Data points are the averages of three separate experiments with three technical replicates each. Error bars correspond to the standard errors of the mean. (C) Images of viability-stained *L. crescens* cells at a selection of time points taken from cultures grown at 29 to 30°C. The scale bar is 50 μm for all images.

In order to determine how the populations of *L. crescens* cells in culture change over time, we performed time-course experiments to determine the living/viable population (P_live_) of the *L. crescens* population and the recoverable population (P_R_) at each growth stage. In previous literature, *L. crescens* was grown at 28°C (8, 14). However, we found that it grows rapidly at 29 to 30°C, with a logarithmic-phase doubling time of 11 to 14 h (Fig. 2A), and thus most liquid culture experiments in this study were performed at 29 to 30°C. To determine whether there were deleterious effects of growth at higher temperature, the *L. crescens* time course experiments were also performed at room temperature (RT; 20 to 22°C under our conditions; Fig. 2B). The P_total_ cell population was derived from the optical density using the number 6E^09^ cells ml^−1^ per OD_600_ unit described above and in Fig. 1. The P_total_ at each time point was subdivided into the living population (P_live_) and the dead population (P_dead_) based on SYTO9/propidium iodide (PI) viability staining. (The calibration curve for the viability staining is shown in Fig. S2.) The P_live_ was further divided into the recoverable population (P_R_) based on the CFU on plates and the “viable but not culturable” population (P_vbnc_; the difference between the P_live_ and the P_R_). An image of 2-week-old colonies on a BM7 plate typical of those used for counting recoverable colonies is shown in Fig. S3.

At 29 to 30°C, the duration of logarithmic growth phase is 4 days (Fig. 2A). The P_live_ at the end of logarithmic phase (3 to 4 days postinfection [dpi]) and the beginning of stationary phase (4 to 5 dpi) is between 80 and 91% of the P_total_ (Fig. 2A and C and Fig. S4A). Although the P_total_ remains stable throughout the stationary phase (5 to 10 dpi), the P_live_ diverges further from the P_total_ at 6 dpi, with only 71% of the population staining as viable (Fig. 2A and Fig. S4A). Also, at 6 dpi the population recoverable as CFU (P_R_) drops precipitously to approximately 7.41% of P_total_ and 11% of P_live_ (Fig. 2A and Fig. S4B). This suggests that in stationary phase a significant fraction of the population remains viable (or at least retains membrane integrity) but cannot resume growth on fresh solid BM7 medium (the P_vbnc_). P_live_ decreases to 41% of P_total_ over 7 to 8 dpi, with little further change over 8 to 10 dpi (Fig. 2A and Fig. S4A). However, the P_R_ continues to drop to an average of 0.1% of P_total_ and 0.3% of P_live_ by 10 dpi, with up to 1,000-fold variability both between replicate cultures and between experiments. Throughout logarithmic phase, the culture becomes more alkaline, with the pH reaching 8.45 by 5 dpi (Fig. 2A). The rapid increase in pH from day 4 to 5 is likely due to the pH rising above 7.5 at 4 dpi, which is the limit of the buffering range of the 55 mM ACES buffer in standard BM7 medium (15). The sharp drop in recoverable cells at 5 to 6 dpi correlates with the pH of the culture reaching 8.5.

The cultures grow more slowly at RT, taking 1.5-fold more time to reach stationary phase (6 dpi rather than 4 dpi at 29 to 30°C). This reduces the alkalinization rate and results in a smaller drop in both P_live_ and P_R_ by 10 dpi compared to cultures grown at 29 to 30°C (Fig. 2A and B; Fig. S4A and B). The RT culture appears to be healthier, with all of the cells viable during logarithmic phase (P_live_ is approximately 100% of the P_total_). The P_R_ is also higher at RT than at 29 to 30°C at similar growth stages (e.g., compare 5 dpi for the 29 to 30°C time course with 7 dpi for the RT time course; Fig. 2 and Fig. S4). Interestingly, the RT cultures at 4 to 7 dpi are the only points in either growth curve at which the recoverable P_R_ is higher than 60% of the P_total_ (Fig. 2B and Fig. S4B). The alkalinization of the RT culture is slower than that of the 29 to 30°C culture, likely due to the lower metabolic rate, but once the pH reaches 8.5, both P_live_ and P_R_ decrease (Fig. 2A and B; Fig. S4A and B). Notably, these data suggest that a pH of 8.5 has an extremely deleterious impact on *L. crescens* recoverability independent of the growth temperature (e.g., compare 6 dpi at 29 to 30°C versus 10 dpi RT).

### Increasing the pH buffering capacity of BM7 medium increases the recoverability of *L. crescens* cells in culture.

**FIG 3:**
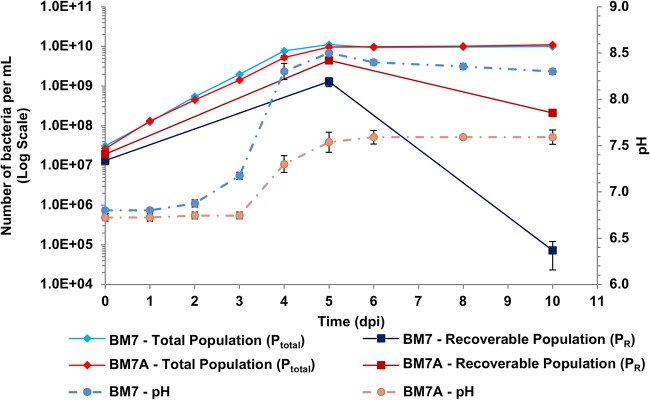
Growth of *L. crescens* at a higher buffer concentration prolongs survival. The total bacterial population (P_total_, diamonds), recoverable bacteria (P_R_, squares), and pH (circles, dotted lines) were determined over 10 days for *L. crescens* cultures grown in standard BM7 (blue) and in BM7 with 110 mM ACES (BM7A medium) (red). The P_total_ and the pH were determined at 0 to 6, 8, and 10 dpi, and the P_R_ was determined at 0, 5, and 10 dpi. Data points are the average of four separate experiments with at least two technical replicates each. Error bars show the standard errors of the mean for all data points.

BM7 medium is buffered by 55 mM ACES, which maintains pH over the range from 6.1 to 7.5 (15). This pH range limit likely explains the increased rate of alkalinization after the cultures reach pH 7.5. To determine whether the pH increase plays an important role in *L. crescens* death during stationary phase, we tested the effect on survival at 30°C of a 2-fold increase (to 110 mM) in medium ACES concentration (BM7A medium, see Table 1). Bacterial growth (P_total_) in BM7 is very similar to growth in BM7A medium (Fig. 3), with a very slight (1.1-fold) but significantly higher 10 dpi density of the BM7A culture (*t* test *P* = 0.032). Although P_total_ changes little, BM7A medium produces a striking improvement in recoverability (P_R_), at 5 dpi (*t* test, *P* = 0.013) and especially at 10 dpi (>2,500-fold increase; *t* test, *P* = 0.0008) (Fig. 3). This correlates with a significantly lower pH in BM7A than in BM7 at 5 dpi and 10 dpi (*t* test, *P* < 0.0001 for both time points) (Fig. 3). These results suggest that much of the rapid decrease in recoverable P_R_ observed in stationary phase in standard BM7 medium is due to the increase in pH.

**TABLE 1:** Names and compositions of growth media

Medium (concn, mM)	αkg (mM)	ACES (mM)	KOH (mM)	FBS (ml)	Grace’s TNM-FH (ml)	NH_4_Cl (mM)	Initial pH
BM7	13.7	55	67	150	300	0	6.8
BM7A	13.7	110	67	150	300	0	6.8
BM7-5dpi-αkg	13.7	55	67	150	300	0	6.8
2× αkg BM7	27.4	55	163	150	300	0	6.8
BANK6.8	27.4	110	128	150	300	18.4	6.8
BANK6.5	27.4	110	104	150	300	18.4	6.5
BAK6.8	27.4	110	128	150	300	0	6.8
BAK6.5	27.4	110	104	150	300	0	6.5
BAN6.8	13.7	110	104	150	300	18.4	6.8
BAN6.5	13.7	110	80	150	300	18.4	6.5
BM7 + NH_4_Cl (4.6)	13.7	55	67	150	300	4.6	6.8
BM7 + NH_4_Cl (9.2)	13.7	55	67	150	300	9.2	6.8
BM7 + NH_4_Cl (18.4)	13.7	55	67	150	300	18.4	6.8
BM7 + NH_4_Cl (36.8)	13.7	55	67	150	300	36.8	6.8
BM7 + NH_4_Cl (73.6)	13.7	55	67	150	300	73.6	6.8
BM7 + NH_4_Cl (147.2)	13.7	55	67	150	300	147.2	6.8

### Increase in culture pH correlates with production of NH_3_/NH_4_^+^.

**FIG 4:**
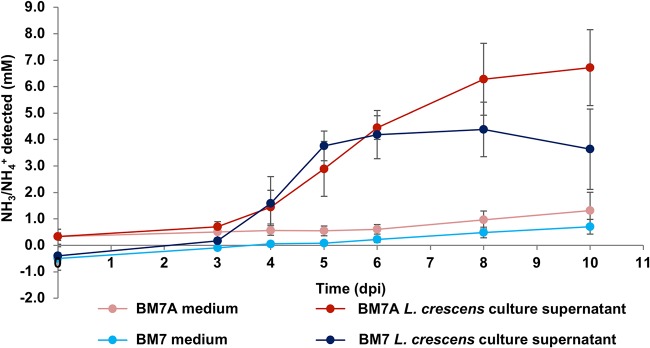
*L. crescens* cultures evolve NH_3_/NH_4_^+^ during growth. The production of NH_3_/NH_4_^+^ over 10-day time courses for *L. crescens* grown in standard BM7 medium (dark blue) and in BM7A (dark red) was determined. The spontaneous production of NH_3_/NH_4_ in uninoculated standard BM7 (light blue) and in uninoculated BM7A (light red) is also shown. *L. crescens* culture data points are averages of four separate experiments with two to three replicates per experiment. Uninoculated medium data points are the average of four separate experiments with one to two replicates per experiment. Error bars represent standard deviations. The millimolar concentrations of NH_3_/NH_4_^+^ produced in BM7 versus BM7A cultures were significantly different at 8 dpi (*t* test, *P* = 0.0042) and at 10 dpi (*t* test, *P* < 0.0001).

The most common cause of medium alkalinization by chemoheterotrophic bacteria grown in complex medium is the evolution of NH_3_ due to deamination of amino acids for use as carbon sources (16). The NH_3_ produced by deamination then deprotonates H_2_O resulting in the generation of OH^–^ ions (17). To determine whether *L. crescens* culture NH_3_ production might play a role in the pH increase of *L. crescens* cultures, the NH_3_/NH_4_^+^ concentration of culture supernatant from standard BM7 medium and from BM7A was determined using the sodium nitroprusside/alkaline hypochlorite method (18, 19). (This assay cannot distinguish between NH_3_ and NH_4_^+^, and thus detected material is referred to as NH_3_/NH_4_^+^.) The results show that NH_3_/NH_4_^+^ is produced by *L. crescens* in both BM7 and BM7A media (Fig. 4). Uninoculated medium appears to accumulate a small quantity of NH_3_/NH_4_^+^, likely from spontaneous breakdown of compounds containing amines (Fig. 4). Production of NH_3_/NH_4_^+^ by *L. crescens* cultures continues several days longer in BM7A medium (110 mM ACES) (to 9 or 10 dpi) than in standard BM7. This suggests that the cells remain metabolically active for a longer period of time in BM7A and is consistent with the survival data shown in Fig. 3. Surprisingly, the maximum concentration of NH_3_/NH_4_^+^ detected in standard BM7 medium is only 4.4 mM. This was unexpected because titration of standard BM7 with NH_3_ from pH 6.8 to the late-stationary-phase pH of 8.23 requires the addition of NH_3_ to a final concentration of 38 mM (Fig. S5A and B). The concentration of NH_3_/NH_4_^+^ detected in the medium after titration demonstrates the accuracy of the sodium nitroprusside/alkaline hypochlorite assays since the values obtained are very close to those expected from the quantity of NH_3_ added (Fig. S5B). The results show that the concentration of NH_3_/NH_4_^+^ detected in the cultures cannot fully explain the large increase in pH. There is more than one possible interpretation of these results. More NH_3_/NH_4_^+^ may have been produced initially but was reassimilated by *L. crescens* cells or was lost due to volatilization. Alternatively, there may be a factor(s) other than NH_3_/NH_4_^+^ production contributing to the increase in culture pH, such as the import of H^+^ ions coupled to the import of organic acids (20, 21).

### Growth of *L. crescens* in BM7 supplemented with NH_4_Cl reduces culture alkalinization.

**FIG 5:**
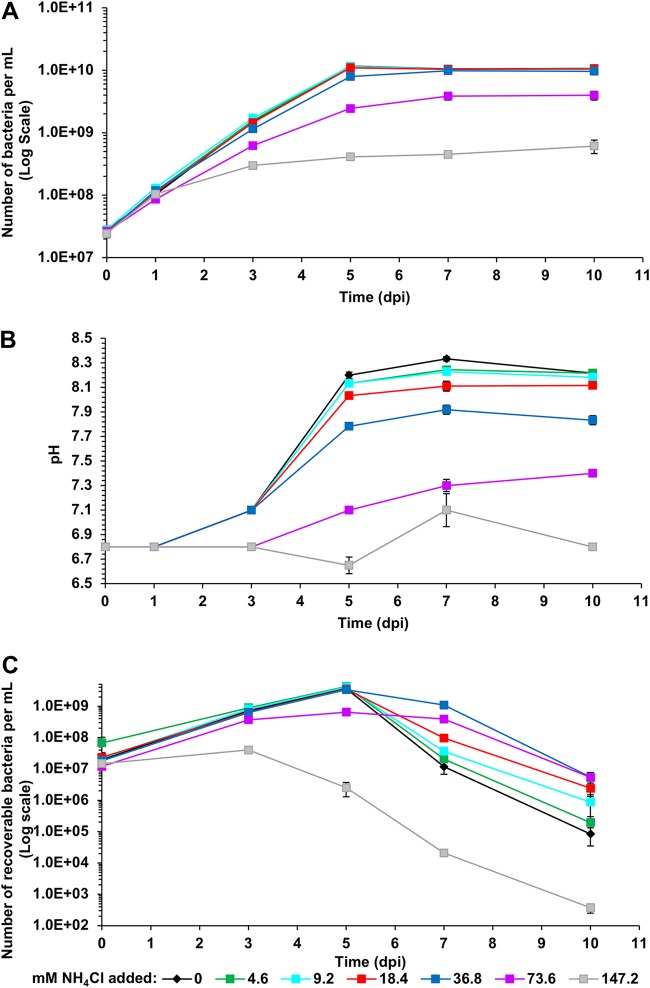
The growth of *L. crescens* in BM7 supplemented with NH_4_Cl enhances survival. The *L. crescens* populations and culture pH in the presence of different NH_4_Cl concentrations (mM) in BM7 medium were assessed. (A) Total population (P_total_); (B) culture pH; (C) recoverable population (P_R_). Growth curves in different NH_4_Cl concentrations are shown in different colors. The graphs show the averages of at least two experiments with three replicates each. Error bars represent the standard errors of the mean.

For some bacteria, high levels of NH_3_/NH_4_^+^ are lethal even if not accompanied by an increase in pH (22). The degree of sensitivity to NH_3_/NH_4_^+^ must be empirically determined for each bacterial species. In order to identify the upper limit of *L. crescens* tolerance to pH-balanced forms of NH_4_^+^ salts, 10-day growth curves were performed on cultures amended with 4.6 to 147.2 mM NH_4_Cl. Ammonium chloride concentrations up to and including 18.4 mM have no effect on *L. crescens* growth (P_total_) (Fig. 5A). At 36.8 mM NH_4_Cl, the growth of the cultures is slightly inhibited (88% of the 2- to 4-day growth rate in standard BM7; analysis of variance [ANOVA], *P* < 0.0001; *post hoc* Tukey honestly significant difference [HSD] test, *P* < 0.0001) and is further inhibited by higher levels of NH_4_Cl (Fig. 5A). At low and intermediate concentrations of NH_4_Cl, an interesting phenomenon was observed—the alkalinization rate was reduced even though there was no growth inhibition. Both 9.2 mM NH_4_Cl (*t* test, *P* = 0.0012) and 18.4 mM NH_4_Cl (*t* test, *P* < 0.0001) cultures have significantly lower pH at 7 dpi of growth than BM7 cultures (Fig. 5B). (Significant differences in pH were determined from *t* tests on the calculated hydronium ion [H_3_O^+^] concentration.) This suggests that the reduced alkalinization rate is not simply due to reduced cell number and consequently to reduced metabolic activity. The presence of NH_4_Cl appears to stabilize the pH. At 7 dpi, the cultures amended with 18.4 to 73.6 mM NH_4_Cl had significantly more recoverable cells (P_R_) than BM7 cultures (*P* ≤ 0.001 from individual *t* tests of each amended medium versus BM7) (Fig. 5C). The increased survival correlates closely with the reduced culture pH. It is important to note that for the 18.4 mM NH_4_Cl culture, this enhanced survival is clearly not due to reduced culture density. Thus, increasing concentrations of NH_4_Cl reduce the culture alkalinization and increase survival. These results also show that there is no deleterious effect of NH_4_Cl concentrations equivalent to or even much greater than the NH_3_/NH_4_^+^ evolved in BM7 cultures (Fig. 4 and 5). (The first evidence of a deleterious effect on survival is at 73.6 mM NH_4_Cl, which has 26.5% survival at 5 dpi compared to 42.6% survival at 36.8 mM NH_4_Cl.) Since 36.8 mM NH_4_Cl is much higher than the maximum 4.4 mM NH_3_/NH_4_^+^ evolved in BM7 medium, this suggests that the accumulation of pH-balanced NH_4_^+^ salts does not play a role in killing of *L. crescens* cultures. We conclude that if the NH_3_/NH_4_^+^ evolved by *L. crescens* growth and detected in the assays shown in Fig. 4 accurately reflects the full amount that is produced, it is highly unlikely that the NH_3_/NH_4_^+^ itself contributes to rapid death in stationary phase. The deleterious effects are almost certainly due to the pH increase.

One possible explanation for NH_4_Cl mitigation of alkalinization is that increased levels of NH_4_Cl could inhibit deamination reactions that lead to rapid pH increase in unamended BM7 media. For example, NH_4_^+^ can inhibit glutamate deamination to alpha-ketoglutarate by the catabolic glutamate dehydrogenase of Streptomyces clavuligerus (23). Addition of NH_4_Cl does not fully abolish the pH increase, suggesting either that deamination reactions producing NH_3_ are not completely inhibited by NH_4_Cl or that other metabolic activities also contribute to the pH increase.

### Raising the alpha-ketoglutaric acid concentration of BM7 increases maximum growth but accelerates alkalinization and cell death.

**FIG 6:**
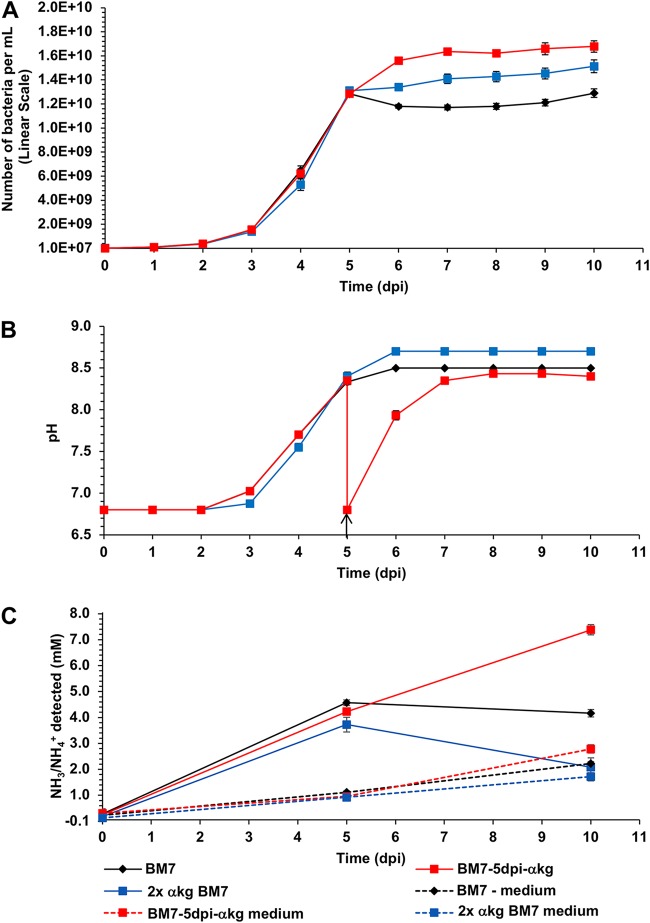
Growth of *L. crescens* with higher α-ketoglutaric acid (αkg) concentrations increases the maximum culture density and increases alkalinization. *L. crescens* growth in BM7 medium was tested with two different modifications to the αkg concentration. First, the amount of αkg was doubled (27.4 mM [2 g liter^−1^]) at culture day 0 (blue line), and second, an additional 2 g liter^−1^ of αkg was added at 5 dpi (red line). Results for standard BM7 are shown in black. (A) Total population (P_total_); (B) pH evolution. The pH reduction observed at 5 dpi is due to the αkg addition (denoted by an arrow in panel B). (C) NH_3_/NH_4_^+^ millimolar concentrations detected in BM7 and BM7 αkg-supplemented *L. crescens* cultures (solid lines) and in the medium controls without bacteria (dotted lines). Error bars represent the standard errors of the mean.

The results described above suggest that the majority of the pH increase is not due to NH_3_/NH_4_^+^ production by *L. crescens*. Therefore, other possible causes of alkalinization were evaluated. Import of protons coupled to the import of organic acids such as alpha-ketoglutaric acid (αkg), the major organic acid carbon source in BM7 medium, would be one such activity (20, 21). As a test of this hypothesis, we altered the concentration of the organic acid carbon source αkg in BM7. In most organisms, the ratio of αkg to glutamate is a key regulatory control point for carbon and nitrogen assimilation reactions (24). If production of NH_3_ is the major driver of the rise in culture pH, increasing the availability of the carbon source αkg would be expected to inhibit or reduce the need for the NH_3_-evolving deamination of amino acids and thus mitigate the alkalinization. If organic-acid associated import of protons is the primary cause of pH rise, an increase in αkg concentration could increase the rate of αkg and H^+^ coimport, exacerbating the alkalinization. We found that doubling the added αkg concentration from 13.7 to 27.4 mM (2× αkg BM7) increased the 7-dpi density of the culture (Fig. 6A) significantly (ANOVA, *P* < 0.0001; *post hoc* Tukey HSD, *P* < 0.0001). This 2-fold increase in the αkg concentration also produced a significantly higher pH of 8.7 by 6 dpi (*t* test, *P* < 0.0001), with no pH change after this time point (Fig. 6B). This suggests that the αkg in BM7 is limiting for biomass production in batch culture. It also suggests that a reaction associated with αkg import drives the pH increase. There is no increase in NH_3_ production associated with the pH increase (Fig. 6C, compare black and blue solid lines). Significantly less NH_3_/NH_4_^+^ is produced at 5 and 10 dpi in 2× αkg BM7 than in BM7 (*t* test, *P* < 0.0001 for both time points). Thus, αkg import rather than NH_3_ production correlates with pH increase. If an additional 13.7 mM αkg (BM7-5dpi-αkg) is added to BM7 cultures at 5 dpi, when the pH had risen above 8, this brings the pH back to 6.8 (marked by arrow in Fig. 6B). Cultures treated in this way reached an even higher density by 7 dpi (ANOVA, *P* < 0.0001; *post hoc* Tukey HSD, *P* < 0.0001) (Fig. 6A, red line). The higher density of the αkg-amended cultures suggests that in BM7, αkg becomes limiting for *L. crescens* growth by 5 dpi as it is consumed. When the pH is adjusted to 6.8 at 5 dpi with 29 mM HCl instead of αkg, there is a smaller increase in culture density (Fig. S6), suggesting that in the αkg-amended cultures, the increase in carbon source drives much of the growth enhancement. The metabolic activity of the BM7-5dpi-αkg culture returns the pH to 8.4 within 2 days of the αkg addition while also evolving NH_3_ (Fig. 6B and C). However, there is only a 1.75-fold increase in NH_3_ between 5 and 10 dpi (Fig. 6C), which is unlikely to be solely responsible for a pH change from 6.8 to 8.4 (a 38-fold decrease in H_3_O^+^ ions). Citric acid has recently been reported to be a better carbon source for *L. crescens* growth than αkg (11); however, under our growth conditions, when an equimolar quantity of citric acid was substituted for αkg in BM7 medium, *L. crescens* had a slightly longer logarithmic phase doubling time compared to that in standard BM7, and the degree of alkalinization at a similar cell density was the same (data not shown).

### Developing new *L. crescens* growth media: improving recoverability and culture yield.

**FIG 7:**
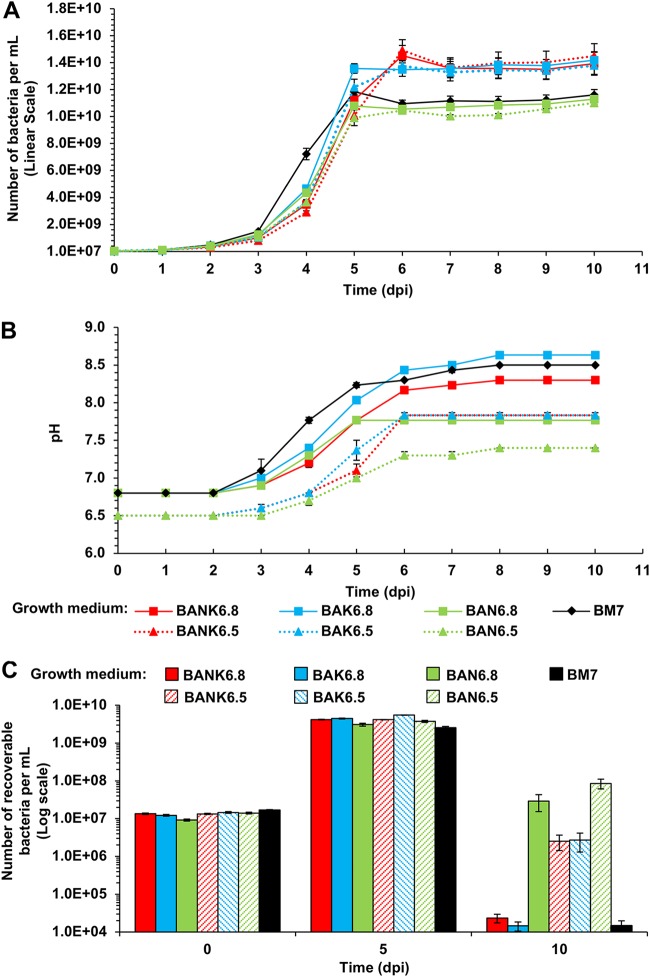
*L. crescens* survival and culture density are improved by modifications to BM7 medium. BM7 medium was amended with different components in order to improve recoverability and to obtain better growth yields for *L. crescens*. The composition of each medium is described in Results and shown in Table 1. (A) Total population (P_total_); (B) pH evolution; (C) recoverable population (P_R_). Error bars represent the standard errors of the mean.

The slow growth and low recoverability of *L. crescens* in the stationary phase in BM7 medium make this species very difficult to study and manipulate. Developing a new medium that increases both total cell yield (P_total_) and recoverability would facilitate its study. Using the results described above, we tested multiple variations of BM7-based medium with the goals of simultaneously improving growth and survival. (Table 1 summarizes the additives in each medium.) For all of these new formulations tested in Fig. 7, double buffering capacity (110 mM ACES) was used to control the rapid alkalinization. The additional variables tested for the ability to mitigate alkalinization were a starting pH of 6.5 instead of 6.8 and the addition of 18.4 mM NH_4_Cl. A starting pH as low as 5.9 has been used for *L. crescens* culture (11), but under our conditions, BM7 medium with a starting pH of ≤6.2 has slower growth (data not shown). The ability of 27.4 mM αkg to increase growth was also tested at pH 6.5 and 6.8 both with and without 18.4 mM NH_4_Cl. (Table 1). *L. crescens* growth, pH, and survival on these media were measured over a 10-dpi period. Overall, none of the new medium formulations improved the logarithmic phase growth rate over that in BM7 (Fig. 7A), but biomass yield and recoverability are improved. The most striking result is that the combined effects of 110 mM ACES and a starting pH of 6.5 allowed the final pH to remain below 8 regardless of the effect of the other medium additives (media BAN-6.5, BAK-6.5, and BANK-6.5) (Fig. 7B). Control of the pH alone improves the recoverability (P_R_) by several orders of magnitude (Fig. 7B and C). If the pH was maintained below 8 with 110 mM ACES and a 6.5 starting pH, the addition of 18.4 mM NH_4_Cl did not provide additional pH control or survival enhancement (Fig. 7B and C, compare BANK-6.5 [red dotted line] to BAK-6.5 [blue dotted line]). Supplementation with 18.4 mM NH_4_Cl is only beneficial if the higher starting pH of 6.8 is used (Fig. 7B and C, compare the red solid line and the blue solid line). *L. crescens* grown in standard BM7 medium has very low recoverability compared to the pH-controlled media (Fig. 7A and C). Growth in media containing 27.4 mM αkg produces the highest stationary-phase *L. crescens* yield (P_total_) (Fig. 7A), with growth continuing until 6 dpi. However, the higher αkg concentration has a deleterious effect on recoverability (Fig. 7C) at 10 dpi due to pH increase (Fig. 7B). This is mitigated if alkalinization is controlled with buffer and a starting pH of 6.5 (Fig. 7C), with no loss of recoverability at 5 dpi. Thus, for optimizing *L. crescens* culture survival we recommend growth in BAN-6.5 (110 mM ACES, a starting pH of 6.5, and 18.4 mM NH_4_Cl) (Fig. 7C, green-striped bar). For optimizing survival and simultaneously enhancing cell yield, we recommend growth in BAK-6.5 (110 mM ACES, a starting pH of 6.5, and 27.4 mM αkg) or BANK-6.5 (110 mM ACES, a starting pH of 6.5, 27.4 mM αkg, and 18.4 mM NH_4_Cl) (Fig. 7A, red and blue dotted lines; Fig. 7C, red- and blue-striped bars). These modifications to BM7 medium provide a considerable advance in culture conditions for *L. crescens*.

## DISCUSSION

Pathogenic bacteria belonging to the *Liberibacter* genus have been responsible for major economic losses in multiple crop species worldwide (10). The HLB-associated bacterium “*Ca*. Liberibacter asiaticus” causes the most devastating citrus disease yet described, with a drop of 75% in citrus production and millions of trees removed in Florida alone (1, 3, 10, 25). The current inability to culture *Liberibacter* pathogens is a major limitation on research and the development of control methods (5, 25). *L. crescens* is the only cultured member of this genus and therefore serves as a critical model organism for the pathogenic *Liberibacter* species (7, 26, 27). *L. crescens* itself is extremely fastidious, and this study details considerable improvements over the standard culture medium. We have also determined several limitations on *L. crescens* growth that may provide insight into the growth requirements of the plant-pathogenic “*Ca*. Liberibacter” species because of the similarities between these bacteria in genes encoding central metabolic functions.

*L. crescens* in this study grew more rapidly and to a higher density than reported in previous studies (7, 8, 11, 26). This increased growth was observed in cultures grown at both 29 to 30°C and at RT (20 to 22°C) and is most likely due to higher aeration on a culture rotator rather than on an orbital shaker (see Materials and Methods). Culturing with higher-aeration conditions in Escherichia coli enhances growth (28). Here, *L. crescens* grown at 29 to 30°C was able to reach an average OD_600_ of 1.5 at 120 h postinoculation and had a doubling time of 11 to 14 h. *L. crescens* at 29 to 30°C had a large difference between the P_live_ and the P_R_ in the stationary phase, with the P_live_ remaining fairly constant from day 7 to 10 but the P_R_ dropping rapidly (Fig. 2A). This state in which bacteria are alive and retain membrane integrity but cannot be recovered in a plating assay is known as viable but not culturable/recoverable (29) (P_vbnc_; see Materials and Methods). This state can be induced by multiple adverse conditions associated with the stationary phase (30). Our results indicate that for *L. crescens* growing in BM7 an increase in pH is responsible for much of the reduced recoverability. However, other stresses cannot be ruled out, including starvation due to the depletion of organic acid carbon sources.

Our results demonstrate that medium alkalinization is one of the most important factors in the stationary-phase death of *L. crescens* in BM7 medium. When the buffering capacity of the medium is doubled (BM7A medium), the H_3_O^+^ ion concentration at 10 dpi is >5-fold higher than in the unmodified BM7, which produces a striking 3,000-fold increase in recoverability (Fig. 3). *L. crescens* cultures evolve NH_3_, which appears to account for some of the pH increase due to production of NH_4_^+^ and the resulting deprotonation of H_2_O to OH^–^ (Fig. 4). However, the low levels of NH_3_/NH_4_^+^ produced by the cultures (Fig. 4) are unlikely to fully account for an increase from pH 6.8 to pH 8.3 to 8.5 (Fig. 2A; Fig. S4B and C). The fact that the addition of high concentrations of NH_4_Cl slightly mitigates pH increase also suggests that NH_3_/NH_4_^+^ production due to deamination reactions is not the primary cause of alkalinization (Fig. 5). Another known cause of medium alkalinization by bacteria is autotrophic fixation of CO_2_ (31). However, this is unlikely to occur in *L. crescens* because it lacks the genes encoding RuBisCO IC for CO_2_ fixation that are present in related proteobacteria such as S. meliloti (32, 33). More likely causes of alkalinization are proton-coupled import of organic acids (20) and/or proton-consuming amino acid and organic acid decarboxylation reactions that in other bacteria are known to lead to medium alkalinization (34). There are many examples of this in the literature. For example, strains of the plant-associated bacterium Azospirillum amazonense have been observed to poison themselves by alkalinizing the medium when grown with the organic acid malate as the carbon source (35). A recent study on E. coli K-12 metabolism shows that growth with sodium succinate or sodium acetate as the sole carbon source in unbuffered medium alkalinizes the medium (21). In Enterococcus faecalis, the malic enzyme encoded by the *maeE* gene is responsible for a malate-consuming reaction that alkalinizes the culture medium (36). In S. meliloti, the NADP^+^-dependent malic enzyme (EC 1.1.1.40) encoded by the *tme* gene catalyzes the oxidative decarboxylation of malate to pyruvate and CO_2_, with the generation of NADPH reductant (37, 38). There is an ortholog of the S. meliloti NADP^+^-dependent malic enzyme in *L. crescens* (B488_RS03960, formerly B488_08280), as well as in “*Ca*. Liberibacter asiaticus” (CLIBASIA_RS00075, formerly CLIBASIA_00080). Another interesting observation from S. meliloti is that moderate medium alkalinization from pH 6.7 to pH 7.1 occurs in unbuffered cultures grown with succinate as the sole carbon source and NH_4_Cl as the sole nitrogen source (39). Significantly, no amino acids were included in these S. meliloti experiments, which means there was alkalinization in the absence of an exogenous supply of amino acids that might lead to NH_3_ production by deamination (39).

Our results suggest that αkg consumption by *L. crescens* is responsible for the majority of the culture alkalinization. Thus, growth of this organism in BM7-based medium may produce such an extreme case of culture alkalinization because both organic acid consumption and NH_3_ evolution drive the culture to a higher pH. αkg is a key regulator of carbon and nitrogen central metabolism, as well as other many other physiological processes (24, 40–42). In some bacteria, αkg limitation can lead to rapid cell death during starvation (42), and our results suggest that αkg may be limiting for *L. crescens* growth. The slow growth of *L. crescens* and the relatively low maximum density it attains may be due to the limited diversity of metabolic functions available in its small genome (43). Strikingly, BM7 medium modifications that slow the alkalinization of *L. crescens* culture such as higher ACES buffer concentration or addition of NH_4_Cl do not permit growth to a higher density. Thus, while the rise in pH has a profound effect on cell survival, it is not the factor that limits the total population growth. The versatile soil bacterium *Sinorhizobium meliloti* 1021 (44) reaches an OD_600_ of >9 in BM7 medium after 72 h growth at 30°C with no detectable pH increase (data not shown). This suggests that S. meliloti 1021 can use a wider array of the nutrient sources available in the very rich BM7 medium and that the ACES in BM7 medium is sufficient to buffer any increase in pH that S. meliloti might produce. The fact that *L. crescens* grows to a higher density when the starting concentration of αkg is doubled (Fig. 6A) suggests that carbon sources in BM7 medium may be limiting for its growth.

Bacterial use of sugars such as glucose as carbon sources can have the opposite effect, to acidify the medium due to acetate secretion (45). This acetate production or “acetogenesis” can occur even under aerobic conditions if the rate of glycolysis/pyruvate dehydrogenase production of acetyl coenzyme A (acetyl-CoA) overcomes the ability of the tricarboxylic acid (TCA) cycle to process the acetyl-CoA (46). Utilization of sugars and amino acids in complex media by E. coli can produce multiple subtle shifts in pH as secretion of acetate and secretion of NH_3_ due to amino acid consumption drive the pH in opposite directions (46). *L. crescens* has a complete glycolytic pathway (8), but the relative contribution of sugar metabolism to *L. crescens* growth remains unclear. It has been demonstrated that sucrose transport dependent upon the product of the *sut* gene occurs in *L. crescens* (47). Relatively strong expression of the genes encoding rate-limiting glycolytic enzymes has also been demonstrated in *L. crescens* (47). The concentration of sucrose in BM7 medium is 23.4 mM, but the extreme alkalinization in *L. crescens* cultures suggests that little or no acetogenesis occurs. It is not yet known how *L. crescens* regulates the catabolism of different carbon sources under different conditions. It is possible that the high concentration of αkg in BM7-based media itself limits *L. crescens* sugar utilization since αkg has been shown in E. coli to exert feedback inhibition on glucose import via the glucose phosphotransferase system (PTS) (40). The *L. crescens* genome encodes components of a PTS (8). The reliance of *L. crescens* on organic acid carbon sources is relevant for the growth of “*Ca*. Liberibacter asiaticus” because “*Ca*. Liberibacter asiaticus” is predicted to have an incomplete glycolytic pathway (8, 47), whereas it has the genes for a complete TCA cycle. If *L. crescens* performs only very limited catabolism of sugars, it may be that at least under some conditions its utilization of carbon sources closely resembles that of “*Ca*. Liberibacter asiaticus.” *Liberibacter* strains have evolved several transporters in order to import nutrients from the environment (8, 25). *L. crescens* BT-1 has a DctA transporter (B488_RS01830, formerly B448_03690) predicted to import C_4_ dicarboxylic acids such as malic acid, succinic acid or αkg, among others (3, 8). The presence of this transporter is consistent with our hypothesis of medium alkalinization through organic acid consumption. The plant-pathogenic “*Ca*. Liberibacter” species also possess this transporter (CLIBASIA_RS01320 in “*Ca*. Liberibacter asiaticus,” formerly CLIBASIA_01360). The “*Ca*. Liberibacter” pathogens also have a dicarboxylate/amino acid:cation symporter (CLIBASIA_RS05230, formerly CLIBASIA_05390), which has 22% identity with an open reading frame in the *L. crescens* genome (B488_RS01530, formerly B448_03060) annotated as a l-cystine transporter and as a member of the sodium/dicarboxylate symporter family. Further work will be required to understand the relative contribution to *L. crescens* metabolism from the consumption of organic acids versus sugars.

The results shown in this study suggest that among the greatest obstacles to the growth of *L. crescens* cultures are the deleterious effects of organic acid consumption in the absence of sufficient control of pH. We recommend that BM7 medium should always be modified with 110 mM ACES buffer, a starting pH of 6.5, and addition of 18.4 mM NH_4_Cl (BAN-6.5) to improve the survival of *L. crescens*. To obtain maximum culture density, growth medium should contain 27.4 mM αkg (BAK-6.5 or BANK-6.5). This study also provides important growth metrics for *L. crescens* that will improve its utility as a model for the plant- and insect-invading *Liberibacter* species and aid in efforts to culture these devastating plant pathogens. These results also highlight the importance of monitoring the evolution of pH even in a well-buffered medium during culture of fastidious organisms.

## MATERIALS AND METHODS

### Strain and growth conditions.

Liberibacter crescens strain BT-1 liquid cultures were maintained in BM7 medium on a TC-7 tissue culture roller (New Brunswick Scientific, Edison, NJ) culture rotator in 25-mm-diameter tubes containing a 5-ml culture volume, set almost horizontally at 45 rpm. BM7 medium includes 13.7 mM α-keto-glutaric acid (αkg), 55 mM ACES buffer (Sigma, A9758; ≥99% titration), 67 mM KOH, 30% TMH-FH insect medium (HiMedia Laboratories), and 15% FBS (VWR-Seradigm), adjusted to pH 6.8. BM7A medium has 110 mM ACES buffer. For BM7 agar plates, Bacto agar (BD) was added to a final concentration of 1.5%. *L. crescens* cultures were maintained at 29 to 30°C over 4 days and reinoculated in fresh BM7 media at an OD_600_ of 0.005. *L. crescens* colonies were maintained on BM7 plates at 27°C for a period up to 30 dpi. All chemicals were from Sigma unless otherwise noted.

### Determination of the number of total bacteria per optical density unit.

We determined the relationship between the OD_600_ and the actual number of bacterial cells per ml by cell counts at multiple culture time points. Bacteria from a logarithmic-growth-phase preculture (4 dpi) were inoculated into fresh BM7 media at a target concentration of 0.005 OD_600_ and grown at 29 to 30°C for 8 days. Samples taken at 0, 2, 4, 6, and 8 dpi were assayed directly from the culture in BM7 media or after centrifugation (15 min at 5,000 × *g*) and resuspension in 0.15 M NaCl at the same concentration as the original culture. Optical density was measured at 600 nm on a Genesys 20 spectrophotometer (Thermo Scientific), and culture dilutions were plated in order to determine the number of recoverable cells in the sample. The total number of bacteria was determined by counting dilutions of both centrifuged and uncentrifuged culture aliquots on an Olympus BX61 microscope at 1,600 magnification with an improved Neubauer phase hemocytometer (Hausser Scientific, Horsham, PA), with two 15-μl drops of each culture deposited onto each of the counting areas. On the microscope, the fine adjustment knob was set at a position in which the Neubauer ruling lines were clearly focused, and then the knob was moved 90° five times in order to cover the space within the slide and the coverslip and count the total number of bacterial cells visible on the hemocytometer grid. Each drop was counted by two different people, with two replicates performed for each of three separate experiments. Calculations for bacteria per ml were performed according to the hemocytometer manufacturer’s instructions. The total number of bacterial cells ml^−1^ per OD_600_ was determined by dividing the total bacterial number ml^−1^ determined from hemocytometer counts (P_total_) by the culture OD_600_. This was determined for both aliquots straight from culture (uncentrifuged) and aliquots that were pelleted and resuspended in 0.15 M NaCl (centrifuged). For the experiments in Fig. 2 in which viability staining was performed, the power equation for centrifuged bacteria (6E^09^*x*^1.0188^) was used to calculate P_total_ from the OD_600_. For the experiments in subsequent figures in which the OD_600_ was measured directly on uncentrifuged cultures, the power equation for uncentrifuged bacteria (6E^09^*x*^0.9901^) was used.

### Viability staining of *L. crescens* cells and calculation of live and dead fractions of the bacterial population.

A Live/Dead BacLight bacterial viability kit (Life Technologies, Eugene, OR) was used to qualitatively and quantitatively determine the live fraction and the dead fraction of the population of *L. crescens* cultures at each time point. When bacteria are alive and have full membrane integrity, SYTO-9 dye is able to penetrate the membrane and stain the DNA, but the larger PI dye molecules are not able to penetrate (Live/Dead BacLight bacterial viability kit L13152 [product information]). PI can penetrate the compromised membrane of dead cells, stain the DNA, overwhelm the emission from the SYTO-9 dye, and therefore stain the cells red. Thus, live cells appear green and dead cells appear red. For the qualitative determination of viability, bacterial cultures centrifuged, and suspended in 0.15 M NaCl were stained with a BacLight 2× stock solution, incubated in the dark at room temperature for 15 min (according to the manufacturer’s protocol), and visualized with an Olympus BX61 fluorescence microscope.

Bacterial viability was quantified by measuring the emission of bacterial suspensions in 0.15 M NaCl stained with the BacLight kit using a SpectraMax M5 microplate reader (Molecular Devices) in top-read mode. The excitation wavelength was 485 nm, and the emission wavelengths were 530 and 630 nm, respectively, for SYTO-9 and PI. Live/dead calibration curves for *L. crescens* BT-1 were performed at known bacterial live/dead ratios at a 0.05 OD_600_ bacterial concentration (Fig. S2). The 530/630 emission ratio was compared to the known live/dead ratio, and the adjusted trend line was used to calculate the percentage of living cells in the bacterial culture. In order to build the calibration curve, 4-dpi bacterial cultures in which 100% of the cells observed by fluorescence microscopy appeared to be living (green) were centrifuged, suspended in 0.15 M NaCl, and split: half of the culture was maintained as the live population, and half was heat killed at 98°C for 90 min. (During the 90-min heat shock, the live half of the population was maintained on the culture rotator.) Cell membrane permeabilization by heat killing of the population was checked by fluorescence microscopy. Living and dead bacterial suspensions were then adjusted to a 0.05 OD_600_ concentration in 0.15 M NaCl. Live and dead cell suspensions were mixed in ratios from 100% live to 0% live in 10% steps. Bacterial live/killed mixes were stained with BacLight 2× stock solution, and six wells from each live/dead percent mix were measured on the microplate reader. The calibration curve was established by plotting the known percentage of live cells in each mixture against the ratio Emission_Syto-9_/Emission_PropidiumIodide_ (Fig. S2). The calibration data were validated by ANOVA (*P* < 0.0001) and a *post hoc* Tukey HSD test (*P* < 0.0001) for all data points. These analyses showed that every percentage tested was significantly different from the others. Therefore, the Emission_Syto-9_/Emission_PropidiumIodide_ ratio estimation (Fig. S2) is valid to determine the P_live_ population in the time course experiments shown in Fig. 2.

### *Liberibacter crescens* growth curve experiments.

*L. crescens* growth experiments were performed under different medium and temperature conditions that are explained below; however, a similar protocol was followed for all of them. Briefly, BM7-media (Table 1) cultures were inoculated at day 0 to a target OD_600_ of 0.005 from logarithmic-phase precultures grown for 4 days at 29 to 30°C in BM7 medium. (The reported 0 dpi value was always derived from the spectrophotometer OD_600_ reading obtained from the newly inoculated culture.) Growth-curve cultures were incubated at 29 to 30°C or room temperature (RT, 20 to 22°C) on a culture rotator for 10 days. Time point samples were subjected to the following analyses: OD_600_, determination of CFU by spot dilutions on BM7 plates, and pH assay (McolorpHast pH strips [EMD Millipore]; see below). For the findings depicted in Fig. 2, BacLight viability assays were also performed. For the experiments in Fig. 2, optical density and plating assays were performed on aliquots taken directly from BM7 cultures (uncentrifuged) and on aliquots that were centrifuged and resuspended in 0.15 M NaCl (centrifuged). Viability staining was only performed on centrifuged cultures due to interference from BM7 autofluorescence. Viability staining was performed only after day 3 to provide a minimum bacterial concentration of 0.05 OD_600_. For the experiments in Fig. 3, 5, 6, and 7, optical density and plating assays were performed on aliquots taken directly from BM7 cultures only (uncentrifuged). Modifications to standard BM7 medium for the experiments in Fig. 3 and 7 are described in Results and in Table 1. pH tests were performed with McolorpHast strips (pH 6.5 to 10; EMD Millipore, catalog number 1.09543.0001), which have 0.2 to 0.3 pH unit graduations in the relevant range. The exception was for Fig. 2, in which pH 6.5 to 10 strip tests were used for one experiment and McolorpHast strips, pH range 5 to 10 (EMD Millipore), which have 0.5 pH unit graduations, were used for the other two separate experiments.

### *Liberibacter crescens* cell population definitions.

The data collected during the *L. crescens* growth experiments were used to define different bacterial populations within the culture. These populations are described below:

### (i) P_total_.

The total population (P_total_) is actual bacterial number (live or dead) present in the culture. Since all of the bacteria participate in light scattering during optical density measurements, this is the number of cells derived from the OD_600_ transformed with the cells/ml/OD_600_ power equation in Fig. 1.

### (ii) P_live_.

The live population (P_live_) is the population calculated to be viable after staining with the BacLight kit. It includes all cells with membrane integrity whether actively multiplying or not (29). This population was quantified by determining the Emission_Syto9_/Emission_PropidiumIodide_ ratio on the fluorescent plate reader and calculating the live/dead percentage from the calibration curve (Fig. S2). The number of live cells was calculated by multiplying the P_total_ by the fraction of live cells.

### (iii) P_R_.

The recoverable population (P_R_) is the population that can grow and form colonies when plated to BM7 medium (CFU). This includes cells that are actively multiplying in the media with a high metabolic rate and those that are not multiplying but are in a reversible “dormant” state, most likely with a low metabolic rate.

### (iv) P_vbnc_.

The viable but not recoverable/culturable population (P_vbnc_) is the population that is alive and participating in nutrient consumption but is unable to divide when plated on fresh BM7 media. These cells have also been described as pseudosenescent (29, 48). The P_vbnc_ is calculated by subtracting the recoverable cells from the living cells, i.e., P_vbnc_ = P_live_ – P_R_.

### pH titration of BM7 medium.

Titration of BM7 with 1 M NH_3_ was performed in order to determine how much NH_3_/NH_4_^+^ is needed to increase the medium pH from 6.8 to 9. Briefly, 1 M solution of NH_3_ was added to BM7 in defined, sequential steps, with the pH measured after NH_3_ step using an AB15 Accumet Basic pH meter (Fisher Scientific). Titrations of BM7 medium were performed four times; the average NH_3_ concentration (in mM) versus pH plot is shown in Fig. S4A.

### Determination of NH_3_/NH_4_^+^ concentration in bacterial cell cultures.

The NH_3_/NH_4_ molar concentration accumulated by *L. crescens* BM7 and BM7A cultures or uninoculated medium was determined using the sodium nitroprusside/alkaline hypochlorite method (Sigma reagents P6994 and A1727, respectively) (18, 19). The protocol was performed as follows. First, 62.5 μl of sample or standard was mixed with (in order) 125 μl of sodium nitroprusside solution, 125 μl of alkaline hypochlorite solution, and 625 μl of Elga system-purified water, followed by incubation for at least 30 min. The optical density of each reaction was determined at a 570-nm wavelength on a Genesys 20 spectrophotometer (Thermo Scientific), with the reaction on a purified water sample set as the blank. The OD_570_ values of 1/25 BM7 culture dilutions in water were compared to a standard curve of NH_4_Cl dilutions made in 1/25 BM7 medium. For BM7A cultures, a standard curve of NH_4_Cl dilutions in 1/25 BM7A was used for comparison. For 2× αkg BM7 (27.4 mM αkg) cultures, a standard curve of NH_4_Cl dilutions in 1/25 2× αkg BM7 was used.

### Statistical analysis.

Data were subjected to statistical tests in order to determine variation in total cell number, pH (as H_3_O^+^ ions), and recoverable cell numbers between the different culture conditions tested. Data from Fig. 5A, 6A, S1C and D, and S2 were subjected to a two-way ANOVA test in which the biological replicate was one of the independent variables. This was followed by a *post hoc* Tukey HSD test to identify significant differences among groups. This method was used to scan the data for variables correlating with differences in growth or in recoverability. The *P* values for differences are reported in Results as the data are described. These analyses were performed using JMP Pro 13.0.0 (Cary, NC). When only two averages were compared, a *t* test was performed. The *P* values for significant differences are reported as the data are described in Results. *t* tests were performed using JMP Pro 13.0.0 or Prism (GraphPad, San Diego, CA).

## Supplementary Material

Supplemental file 1
