# Supplementary material for: Growth Dynamics and Survival of Liberibacter crescens BT-1, an Important Model Organism for the Citrus Huanglongbing Pathogen “Candidatus Liberibacter asiaticus”
Source: Appl Environ Microbiol. 2019 Oct 16;85(21):e01656-19. doi: 10.1128/AEM.01656-19 (PMC6803310; doi:10.1128/AEM.01656-19)
Supplement: Supplemental file 1 [file AEM.01656-19-s0001.pdf]

Figure S1

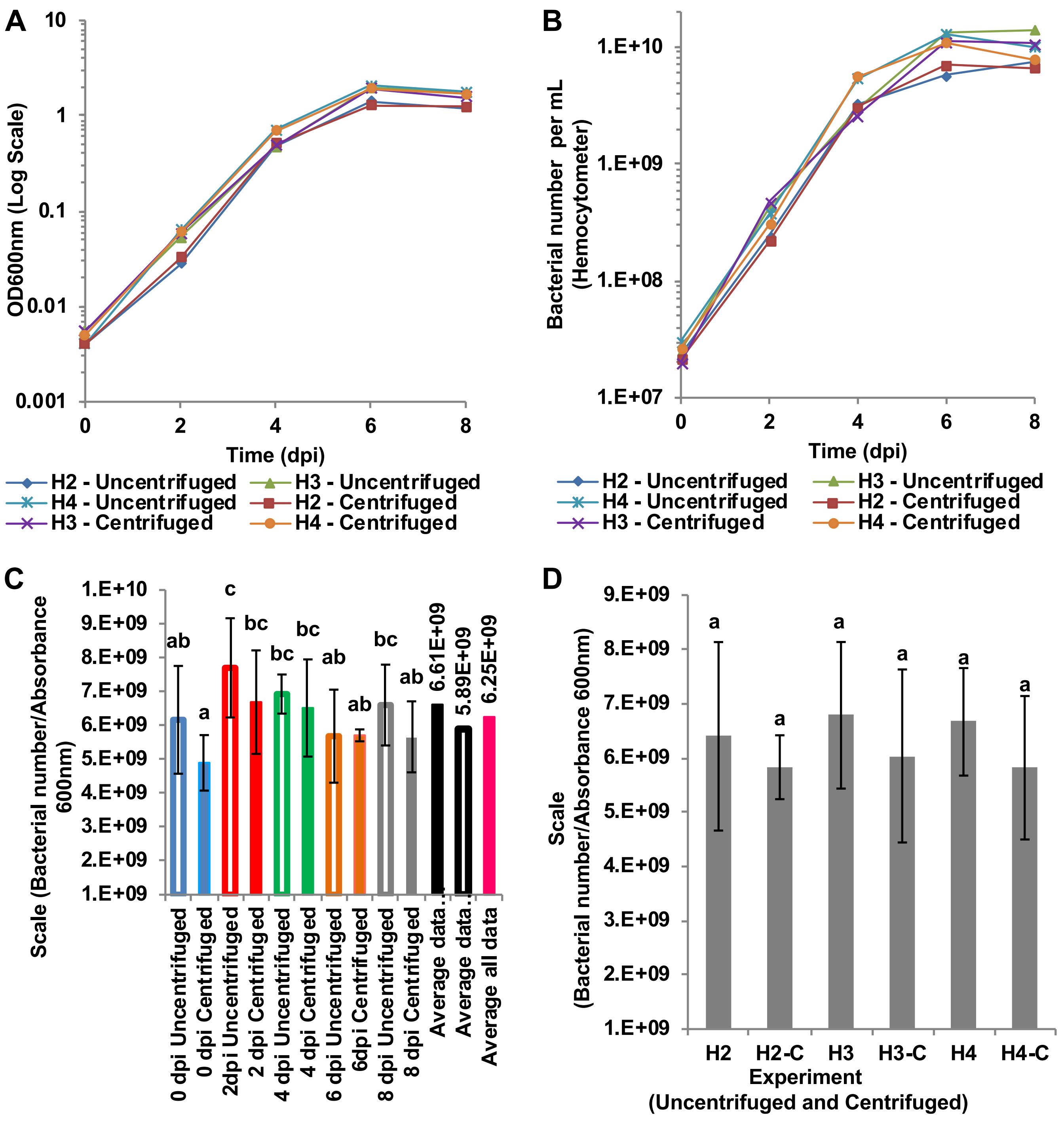

Supplemental Figure 1. Analysis of *L. crescens* hemocytometer counting experiments.

The data from hemocytometer counts of *L. crescens* were analyzed in several ways. (A) *L. crescens* growth curve showing the optical density on the y-axis for each of three different experiments (H2, H3, H4) on a logarithmic scale. (B) *L. crescens* growth curve showing the number of bacteria counted on the hemocytometer (y-axis) for the same experiments (H2, H3, H4). (C) Plot of the average number of bacteria per OD_{600} for each time point shows that although there is variation between data points, the variation is not dependent on the growth stage of the culture. Statistically different groups are denoted by different letters obtained from ANOVA analysis (P <0.0001) and separated by *post hoc* Tukey HSD tests (P values range from 0.0363 to <0.0001).(D) Plot of the average number of bacteria per OD_{600} for each of the three experiments, with all time points averaged together, showing no variation between experiments (ANOVA P = 0.98).

Figure S2

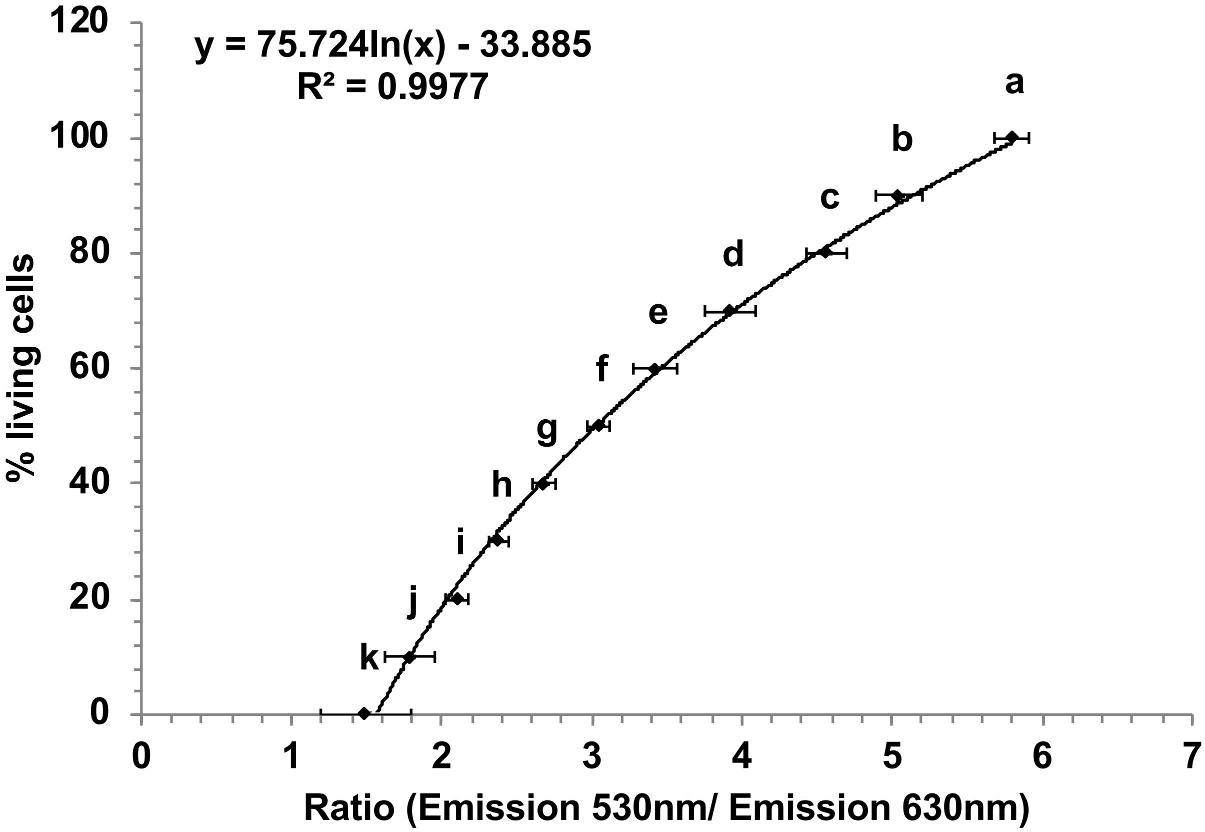

Supplemental Figure 2. Calibration of the *L. crescens* live/dead ratio to the SYTO-9/PI fluorescence emission ratio.

The percentage of live *L. crescens* in defined mixtures of live and heat-killed bacteria is plotted against the ratio of SYTO-9 fluorescence emission (530 nm, live bacteria)/Propidium iodide (PI) emission (630 nm, dead bacteria). A calibration curve based on the percentage of live *L. crescens* vs. the emission ratio was produced from this graph. Values correspond to the mean of two different experiments with at least three technical replicates each. Error bars correspond to the standard deviation from all replicates performed (technical and biological). The logarithmic equation in the graph shows the best match trendline fitting the data. Data were subjected to ANOVA and HSD Tukey analyses. The letter over each data point indicates separation into a statistically different group by ANOVA analysis (P <0.0001) and *post hoc* Tukey HSD tests (P <0.0001).

Figure S3

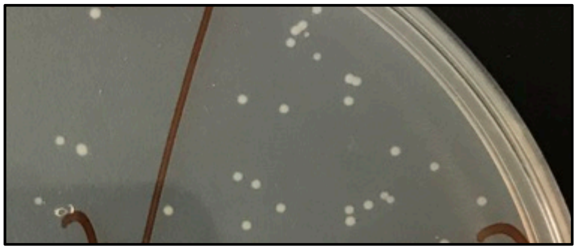

2-week-old *L. crescens* colonies on BM7 agar

Supplemental Figure 3. Representative image of *L. crescens* colonies. BM7 medium plate with dilutions of *L. crescens* colonies grown for 2 weeks. Two-week-old dilution plates were routinely used to count recoverable cells from cultures. The majority of colonies were uniform in growth rate and appearance.

Figure S4

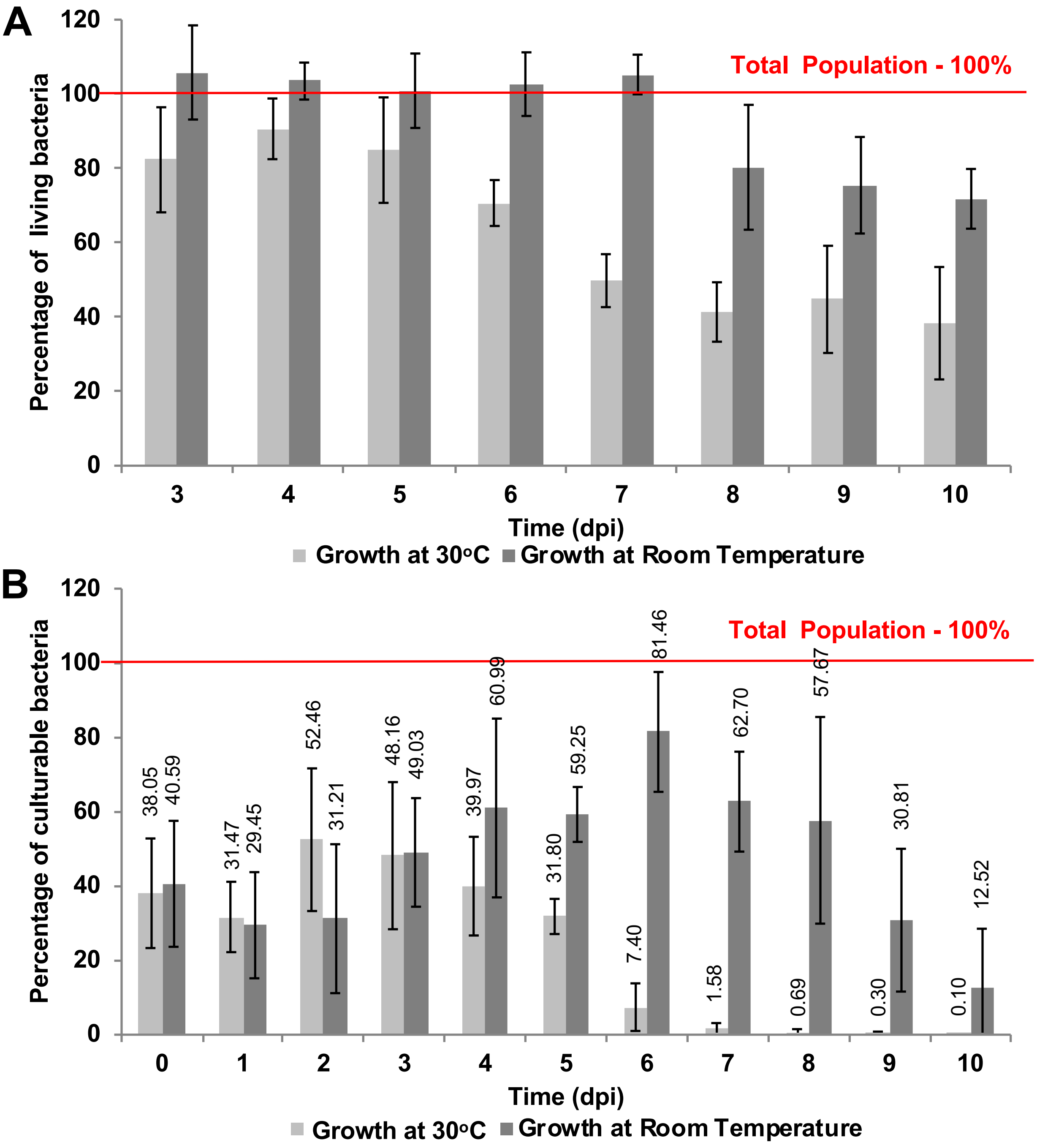

Supplemental Figure 4. Percentages of living and recoverable bacteria in *L. crescens* cultures.

For *L. crescens* cultures at each day of a 10-day timecourse, the graph shows the percentage of living bacteria, derived from the viability staining (A), and the percentage of recoverable bacteria, derived from dividing P_R/P_{total} (B). The total cell population of *L. crescens*, P_{total}, was set as 100% (red line). Light grey bars show the percent live of 29-30°C cultures and dark grey bars show the percent live of RT cultures. See Materials and Methods for viability stain details. The data is the average of three experiments and the error bars represent the standard deviation. In a culture with 100% live bacteria, the live/dead assay shows that all cells have intact membranes. We hypothesize that the percent live is slightly higher than 100% for some time points at RT because the live/dead calibration curve was performed on cells grown at 29-30°C and this might affect the way cells are stained by SYTO-9 and propidium iodide in the viability assay (see Materials and Methods).

Figure S5

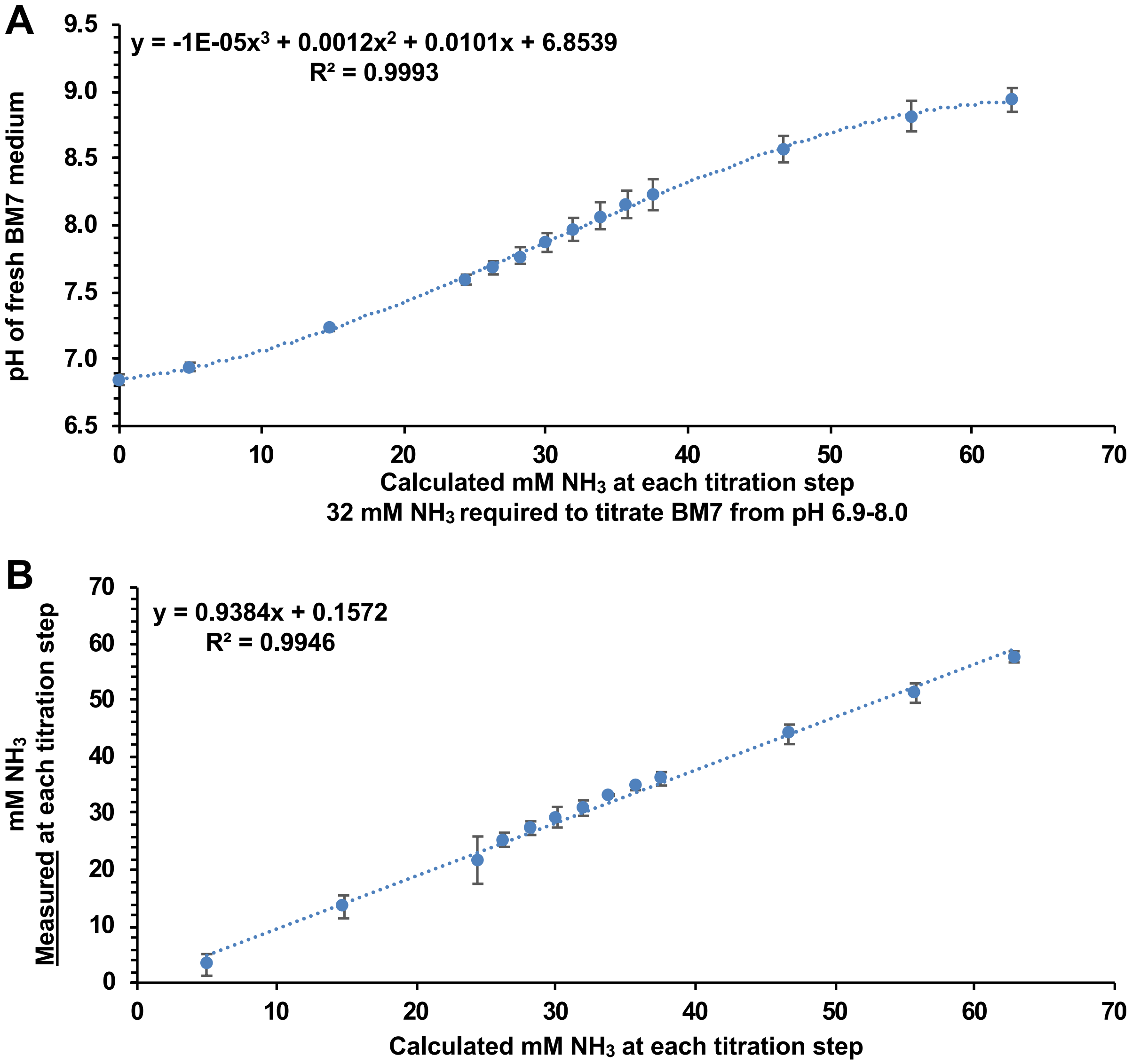

Supplemental Figure 5. BM7 medium titration with NH_3.
(A) NH_3 mM concentration required to titrate standard BM7 medium containing 54.89 mM ACES buffer to pH 9. Graph shows the average of four experiments. The best fit equation and the R^2 value are shown. (B) The NH_3 concentration at each step in the titration was determined using the sodium nitroprusside/alkaline hypochlorite method (see Materials and Methods). Graph shows the average of 2 experiments. The results fit in a linear equation with the linear factor close to 1 and the constant close to 0. This demonstrates the ability of the sodium nitroprusside/alkaline hypochlorite method to accurately detect the quantity of added NH_3 at each step in the titration.

Figure S6

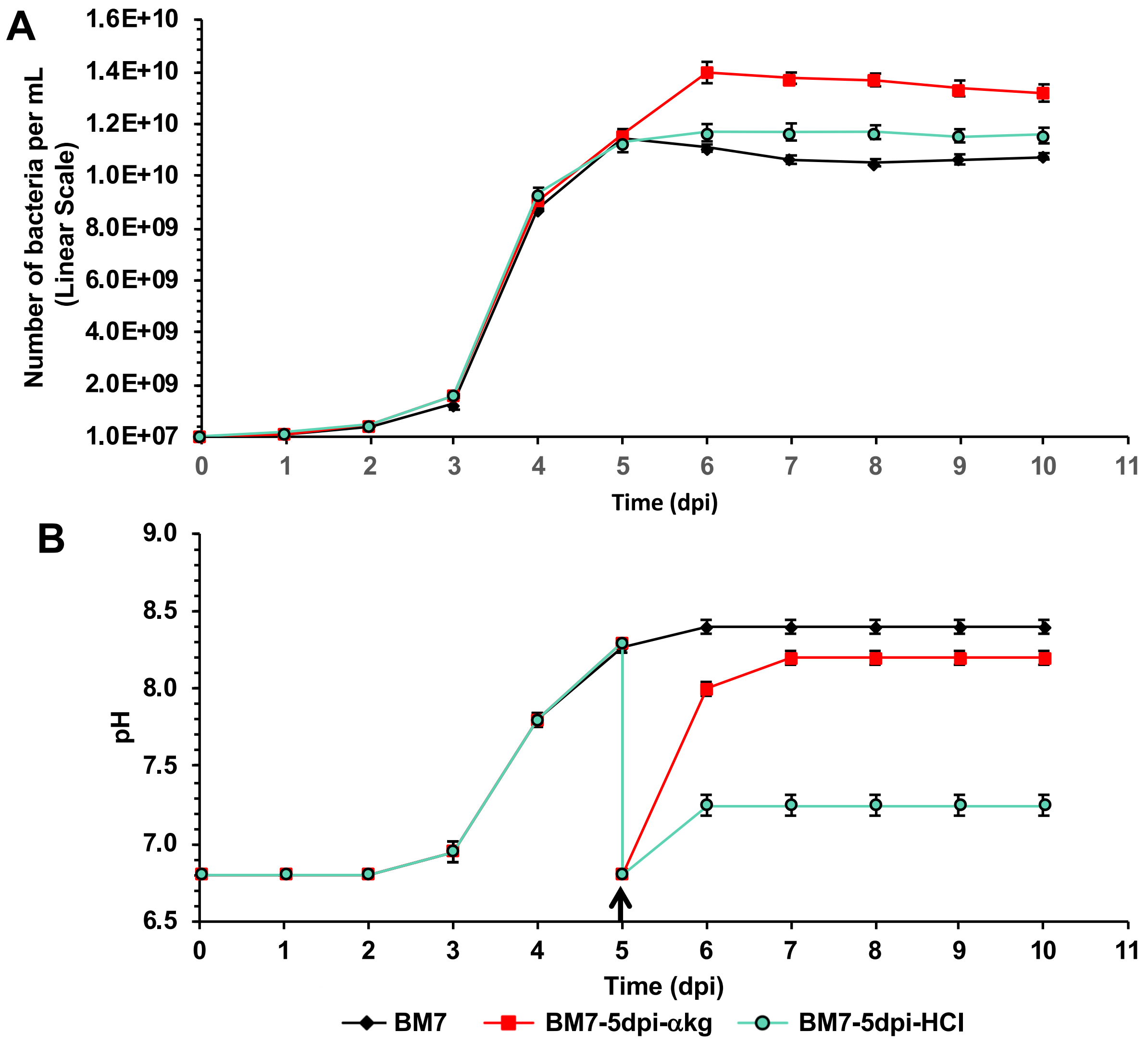

Supplemental Figure 6. Adjustment of 5 dpi *L. crescens* cultures to pH 6.8 with 29 mM HCl does not strongly enhance growth.
L. crescens was grown in BM7 medium without any pH adjustment at 5 dpi (black lines). It was also grown with an additional 2 gr L^{-1} of αkg added at 5 dpi (red lines), which drives the pH down to 6.8. The effect of pH-amendment to 6.8 with 29 mM HCl was also tested (green lines). (HCl reduces the pH without adding additional carbon source.) (A) Total population, P_{total}; (B) pH change. (The pH reduction at 5 dpi due to αkg or HCl addition is denoted with an arrow in B. Error bars represent the standard error of the mean.
